# PSMA-targeted PET imaging for brain metastases from non-prostatic solid tumors: a systematic review

**DOI:** 10.3389/fonc.2025.1553505

**Published:** 2025-03-17

**Authors:** Sara Dall’ Armellina, Gayane Aghakhanyan, Alessio Rizzo, Salvatore C. Fanni, Giacomo Aringhieri, Lorenzo Faggioni, Dania Cioni, Emanuele Neri, Duccio Volterrani, Silvia Morbelli

**Affiliations:** ^1^ Nuclear Medicine Unit, Service Area Department, Azienda Socio-Sanitaria Territoriale (ASST)-Rhodense P.O. Bollate, Milan, Italy; ^2^ Department of Translational Research and of New Surgical and Medical Technology, Nuclear Medicine Unit, University of Pisa, Pisa, Italy; ^3^ Department of Nuclear Medicine, Candiolo Cancer Institute, Fondazione del Piemonte per l'Oncologia - Istituto di Ricovero e Cura a Carattere Scientifico (FPO-IRCCS), Turin, Italy; ^4^ Department of Translational Research and of New Surgical and Medical Technology, Academic Radiology, University of Pisa, Pisa, Italy; ^5^ Department of Medical Sciences, University of Turin, Turin, Italy; ^6^ Azienda Ospedaliero-Universitaria Città della Salute e della Scienza di Torino, Nuclear Medicine Unit, Turin, Italy

**Keywords:** brain metastases, neuro-oncology, non-prostatic solid tumors, Positron-Emission Tomography, PSMA PET, theranostics

## Abstract

**Introduction:**

Prostate-Specific Membrane Antigen (PSMA) is a transmembrane glycoprotein initially identified in prostate cancer (PCa) but also expressed in the neovasculature of various solid tumors. Recently, PSMA PET has emerged as a promising tool for detecting brain metastases (BMs) from non-prostatic cancers, offering diagnostic capabilities in addition to conventional imaging. This systematic review evaluates the role of PSMA-targeted radiopharmaceuticals in imaging BMs, highlighting their comparative diagnostic performance and exploring their potential for theranostic applications.

**Methods:**

A systematic review of the literature was conducted following PRISMA guidelines. Studies evaluating the diagnostic accuracy of PSMA PET imaging in identifying brain metastases (BMs) from non-prostatic solid tumors were included. Both full research articles and case reports were considered to capture the breadth of current evidence. The methodological quality of the included studies was assessed using the QUADAS-2 tool, and data were synthesized qualitatively.

**Results:**

The review includes 23 studies reporting on 77 BMs from diverse primary malignancies, including lung, breast, salivary gland, thyroid, kidney, and melanoma. PSMA PET demonstrated high tumor-to-background ratios (TBR), enabling superior detection of BMs compared to conventional imaging modalities such as contrast-enhanced MRI and [18F]FDG PET. In post-radiotherapy cases, PSMA PET effectively differentiated radionecrosis from tumor recurrence. Moreover, PSMA PET demonstrated superior sensitivity in detecting thyroid metastases compared to traditional scintigraphy methods, highlighting its potential in cases where standard techniques yield inconclusive results.

**Conclusions:**

PSMA PET imaging shows significant promise in improving the diagnosis and management of BMs from non-prostatic cancers. While its theranostic applications remain underexplored, initial findings suggest promising avenues for integrating PSMA PET into personalized neuro-oncology care. Future studies should focus on standardizing imaging protocols, exploring PSMA PET utility in diverse tumor subtypes, and validating its role in clinical decision-making to maximize its impact on patient outcomes.

## 1 Introduction

Prostate-Specific Membrane Antigen (PSMA) is a transmembrane glycoprotein overexpressed in prostate cancer (PCa) cells, making it an essential biomarker in oncologic imaging and targeted therapy (1). Recent advances in PSMA-targeted imaging, particularly with positron emission tomography (PET) tracers, have revolutionized diagnostics and staging for PCa. Tracers such as [68Ga]PSMA-11 and [18F]PSMA-1007 have demonstrated exceptional sensitivity and specificity, enabling the detection of early metastases and residual disease, even at low prostate-specific antigen levels (2, 3).

Beyond prostate cancer, PSMA expression has been observed in the neovasculature of various non-prostatic solid tumors, including brain metastases (BMs) (4, 5). Primary cancers such as those of the breast, lung, skin (melanoma), colon, and kidneys frequently metastasize to the brain making BMs a leading cause of cancer-related mortality (6). BMs, which are significantly more common than primary central nervous system (CNS) tumors, are often challenging to diagnose and manage due to their location and the limitations of conventional imaging modalities. Contrast-enhanced magnetic resonance imaging (CE-MRI) and computed tomography (CE-CT) are currently the standard imaging techniques for CNS lesions. Still, they have limitations, such as difficulty distinguishing radionecrosis from tumor recurrence or reduced utility in patients with contraindications to contrast agents (6, 7).

PET imaging, particularly with PSMA-targeting tracers, has emerged as a valuable complementary modality by detecting functional changes earlier than anatomical changes and providing whole-body imaging without the restrictions of metallic implants or renal impairment. Additionally, PSMA tracers exhibit minimal physiological uptake in the brain, enabling superior target-to-background ratios (7, 8). [18F]Fluorodeoxyglucose ([18F]FDG) remains the most widely utilized radiopharmaceutical in oncology; however, its application in brain imaging is limited due to high physiological uptake in normal brain tissue, as well as non-specific uptake in inflammatory and infectious conditions. Alternative tracers, such as [18F]fluoroethyltyrosine ([18F]FET), [18F]dihydroxyphenylalanine ([18F]DOPA), and [11C]methionine, have demonstrated superior diagnostic performance for brain tumors. Despite their advantages, these tracers, like [18F]FDG, lack the capability for theranostic applications, restricting their utility to diagnostic purposes alone (8, 9).

The role of PSMA PET imaging in BMs from non-prostatic tumors is an area of growing interest. The current literature concerning the employment of PSMA-targeting in non-prostatic solid tumors BMs is primarily limited to isolated cases or small case series. Many of these studies have highlighted incidental findings of BMs that were not detected by conventional imaging, demonstrating the potential of PSMA-targeted imaging to influence clinical management (10–12). Although evidence remains limited, emerging prospective studies are beginning to validate its diagnostic utility and explore its theranostic applications in non-prostatic oncology, particularly for challenging neuro-oncological cases with lesions that are difficult to treat with conventional methods (13–15).

This review aims to evaluate the potential of PSMA-targeting radiopharmaceuticals to assess CNS metastases from non-prostatic cancers. The objective is to synthesize existing literature to explore the potential of PSMA tracers for theranostic applications, with the goal of advancing personalized neuro-oncology care.

## 2 Methods

This systematic review was registered in the PROSPERO database and conducted according to the Preferred Reporting Items for Systematic Reviews and Meta-Analyses (PRISMA) guidelines. We performed a literature search across five major databases: PubMed, Embase, Scopus, Google Scholar, and the Cochrane Library, including studies published up until December 2024. The search strategy utilized combinations of MeSH terms and keywords such as ((“Central Nervous System Neoplasms”[MeSH] OR “Central Nervous System” OR “Brain Neoplasms”[MeSH] OR “Brain Tumors” OR “Brain Metastases”) AND (“Positron-Emission Tomography”[MeSH] OR “PET”) AND (“Prostate-Specific Membrane Antigen” OR “PSMA”)). No language restrictions were applied. In several studies, subsets of patients with BMs were identified, even though these metastases were not the primary focus of the research, and case report e case series were included.

In line with the review’s objective, clinical studies reporting, the diagnostic accuracy of PSMA-targeting radiopharmaceuticals in detecting BMs of the CNS were selected, when available. Abstracts were reviewed by the authors, who independently decided which studies to include or exclude based on their relevance to the review’s focus, aiming to minimize potential selection bias. Any reviewer discrepancies between the reviewers were resolved through online meeting to reach a consensus.

Duplicate records were removed using Rayyan, an AI-powered platform for systematic reviews (https://www.rayyan.ai/).

Quality assessment of included studies was performed using the Quality Assessment of Diagnostic Accuracy Studies-2 (QUADAS-2) tool. This tool evaluates four domains: patient selection, index test, reference standard, and flow and timing (**Table 1**); a single answer “no” response in a domain resulted in high risk of bias.

**Table 1:** Bias risk and concerns sources.

Criteria	Answer
Domain 1: Patient selection (Risk of Bias)	
Was a consecutive or random sample of patients enrolled?	Yes/No/Unclear
Was a case-control design avoided?	Yes/No/Unclear
Did the study avoid inappropriate exclusions?	Yes/No/Unclear
Could the selection of patients have introduced bias?	RISK: Low/High/Unclear
Domain 1: Patient selection (Applicability)	
Is there concern that the included patients do not match the review question?	CONCERN: Low/High/Unclear
Domain 2: Index test (Risk of Bias)	
Were the index test results interpreted without knowledge of the results of the reference standard?	Yes/No/Unclear
If a threshold was used, was it pre-specified?	Yes/No/Unclear
Could the conduct or interpretation of the index test have introduced bias?	RISK: Low/High/Unclear
Domain 2: Index test (Applicability)	
Is there concern that the index test, its conduct, or interpretation differ from the review question?	CONCERN: Low/High/Unclear
Domain 3: Reference standard (Risk of bias)	
Is the reference standard likely to correctly classify the target condition?	Yes/No/Unclear
Were the reference standard results interpreted without knowledge of the results of the index test?	Yes/No/Unclear
Could the reference standard, its conduct, or its interpretation have introduced bias?	RISK: Low/High/Unclear
Domain 3: Reference standard (Applicability)	
Is there concern that the target condition as defined by the reference standard does not match the review question?	CONCERN: Low/High/Unclear
Domain 4: Flow and timing (Risk of bias)	
Was there an appropriate interval between index test(s) and reference standard?	Yes/No/Unclear
Did all patients receive a reference standard?	Yes/No/Unclear
Did patients receive the same reference standard?	Yes/No/Unclear
Were all patients included in the analysis?	Yes/No/Unclear
Could the patient flow have introduced bias?	RISK: Low/High/Unclear

## 3 Results

**Figure 1:**
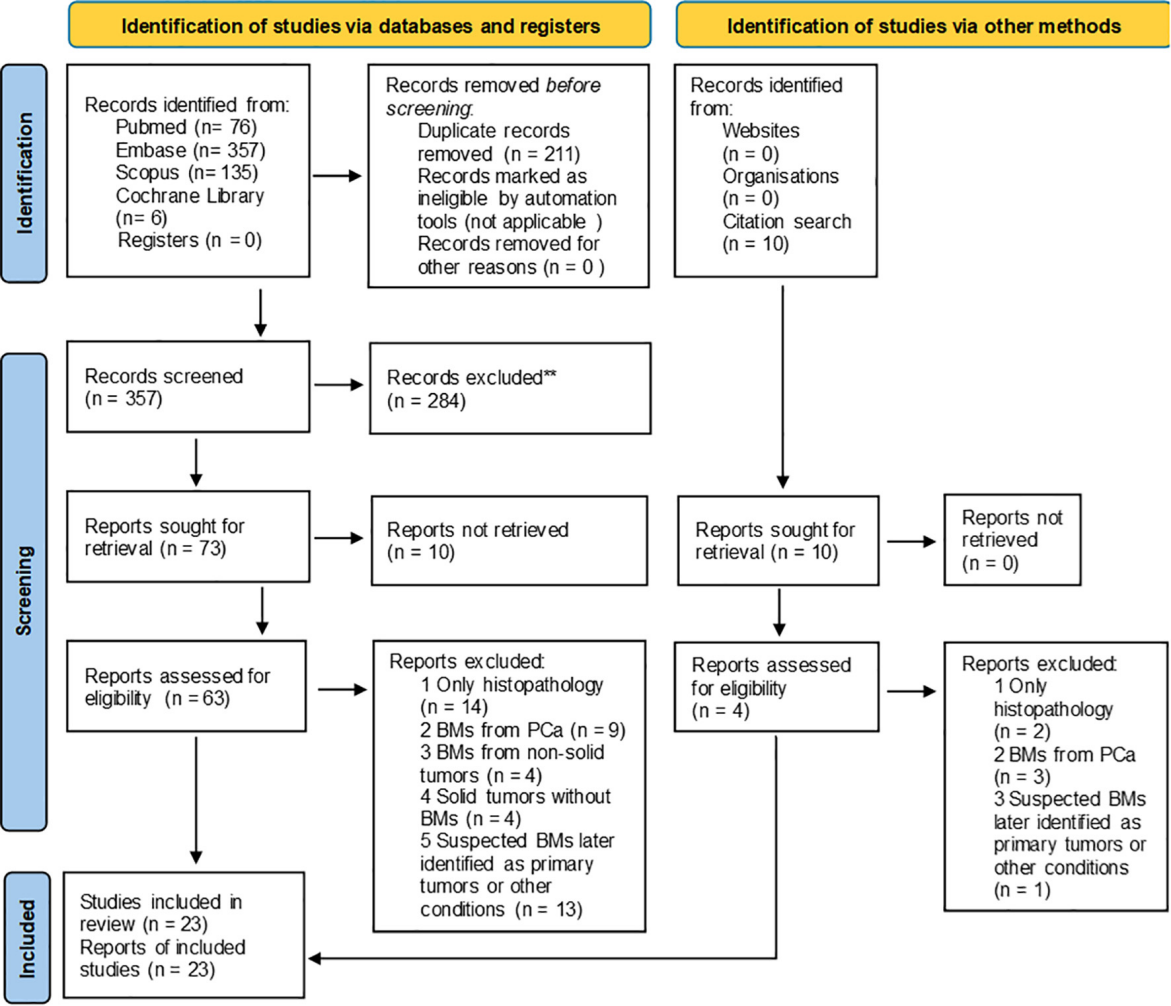
PRISMA flowchart of research strategy and study selection.

**Figure 2:**
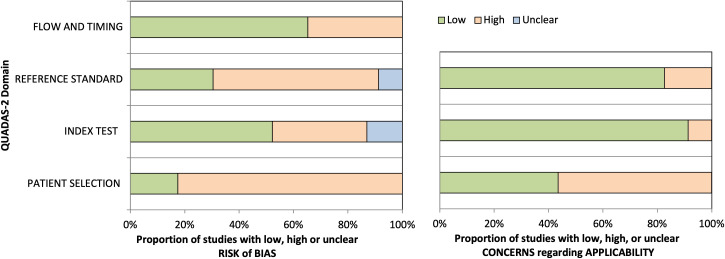
Graphical display for QUADAS-2 results.

Of the 574 retrieved articles, 211 duplicates were eliminated, leaving 357 unique records for screening. After reviewing titles and abstracts, 73 articles were selected for further consideration. Reference lists of these articles were examined to identify additional relevant studies, resulting in a final selection of 23 articles that met the inclusion criteria (**Figure 1**). Full-text versions were downloaded for all included articles, except of three. The QUADS-2 results were synthesized in a tabular format (**Table 2**) and visually presented in graphs (**Figure 2**) to indicate risk of bias and applicability concerns.

**Table 2:** Tabular presentation for QUADAS-2 results.

Study	RISK OF BIAS				APPLICABILITY CONCERNS		
	PATIENT SELECTION	INDEX TEST	REFERENCE STANDARD	FLOW AND TIMING	PATIENT SELECTION	INDEX TEST	REFERENCE STANDARD
Study 1							
Study 2			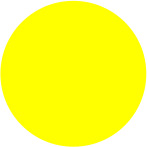				
Study 3			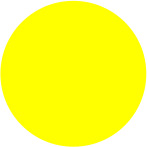				
Study 4							
Study 5		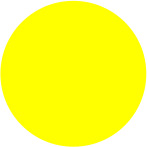					
Study 6							
Study 7							
Study 8							
Study 9							
Study 10							
Study 11							
Study 12							
Study 13		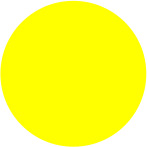					
Study 14		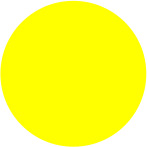					
Study 15							
Study 16							
Study 17							
Study 18							
Study 19							
Study 20							
Study 21							
Study 22							
Study 23							

Legend: red circle – high risk; yellow circle – intermediate risk; green circle – low risk

Some questions were not applicable to case reports; in such cases, the response was marked as “unclear.”

The 23 selected articles include 46 patients with 77 brain metastases (BMs) originating from various primary malignancies: lung cancer (23 BMs), breast cancer (17 BMs), salivary gland tumors (16 BMs), thyroid carcinoma (10 BMs), kidney cancer (8 BMs), and melanoma (3 BMs). Only one was counted in cases where the number of BMs was unspecified.

Most studies focused on BMs from breast and lung cancers, with 6/23 articles addressing breast cancer and 5/23 addressing lung cancer. Similarly, BMs from these two cancer types accounted for a significant proportion of the total: 17/77 from breast cancer and 22/77 from lung cancer.

PSMA-targeting radiopharmaceuticals varied across studies. [68Ga]-labeled PSMA radioligands were used in 60% of cases (14/23), with [68Ga]PSMA-11 used in 6/23 studies and an unspecified [68Ga]PSMA tracer in the remaining cases. [18F]-labeled tracers were used in 31% of cases (7/23), including [18F]PSMA-1007 (5/23) and [18F]DCFPyL (2/23). Other radiopharmaceuticals included [89Zr]Df-IAB2M anti-PSMA minibody and [18F]AlF-PSMA-11, each used in one case (**Table 3**).

**Table 3:** Summary of article publication year, number of patients and lesions, type of primary tumor, population characteristics, radiopharmaceutical used and co-registration imaging modality.

Authors	Year	N° patient	N° lesion	Primary tumors	Age (Years)	Sex	Radiopharmaceutical	Hybrid Imaging
Rowe et al. (32)	2015	1	1	Kidney	61	M	[18F]DCFPyL	PET/CT
Taywade et al. (17)	2016	1	5	Thyroid	50	M	[68Ga]PSMA-11	PET/CT
Medina- Ornelas et al. (30)	2017	1	1	Breast	45	F	[68Ga]PSMA*	PET/CT
Matsuda et al. (26)	2018	1	1	Lung	54	M	[89Zr]Df-IAB2M anti-PSMA Mb	PET/MRI
Malik et al. (18)	2018	1	1	Breast	37	F	[68Ga]PSMA*	PET/CT
Raveenthiran et al. (33)	2019	1	1	Kidney	N.A.	N.A.	[68Ga]PSMA-11	PET/CT
Filizoglu et al. (34)	2019	1	3	Kidney	43	M	[68Ga]PSMA*	PET/CT
Yin et al. (35)	2019	1	3	Kidney	58	M	[18F]DCFPyL	PET/CT
Marafi et al. (19)	2020	1	3	Breast	64	F	[18F]PSMA-1007	PET/CT
Hod et al. (37)	2020	1	1	Melanoma	76	M	[68Ga]PSMA*	PET/CT
van Boxtel et al. (40)	2020	1	13	Salivary Glands	N.A.	N.A.	[68Ga]PSMA-11	PET/CT
Pitaula-Cortes et al. (36)	2021	2	3	Thyroid	52-63	F	[68Ga]PSMA-11	PET/CT
Arslan et al. (20)	2021	1	1	Breast	47	F	[68Ga]PSMA-11	PET/CT
Datta Gupta et al. (21)	2021	1	1	Salivary Glands	45	F	[68Ga]PSMA*	PET/CT
Mehdi et al. (38)	2022	1	N.A.	Melanoma	N.A.	N.A.	[18F]PSMA-1007	PET/CT
Pei et al. (27)	2023	7	N.A.	Lung	43-76	M	[68Ga]PSMA-11	PET/CT
Lattuada et al. (28)	2023	11	15	Lung	N.A	N.A	[68Ga]PSMA*	PET/CT
Dall’Armellina et al. (29)	2023	1	2	Lung	78	M	[18F]PSMA-1007	PET/CT
Shamim et al. (22)	2023	2	2	Salivary Glands	N.A.	N.A.	[68Ga]PSMA-11	PET/CT
Andryszak et al. (23)	2024	1	10	Breast	48	F	[18F]PSMA-1007	PET/CT
Pruis et al. (31)	2024	1 4	1 4	Breast Lung	63 57-70	F M	[68Ga]PSMA-11	PET/MRI
Van den Broeck et al. (24)	2024	2	2	Thyroid	75 59	M F	[18F]AlF-PSMA-11	PET/CT
Mendanha et al. (39)	2024	1	N.A.	Melanoma	50	M	[18F]PSMA-1007	PET/CT

*Unspecified type of radiopharmaceutical.

The diagnostic performance of PSMA PET was compared to other imaging modalities in most studies: contrast-enhanced MRI in 74% (17/23), contrast-enhanced CT in 9% (2/23), [18F]FDG PET/CT in 39% (9/23), and [131I] scintigraphy with therapeutic activity in 13% (3/23). PSMA PET achieved a 100% detection rate for all identified BMs except for one lesion, which exhibited equivocal uptake (SUVmax 0.5). MRI also detected all BMs, while CT identified BMs in only 1 of 2 cases. Notably, [18F]FDG PET/CT failed to detect BMs in 12 out of 24 cases. In patients with BMs from thyroid carcinoma, [131I] scintigraphy identified only 1 of 10 metastases (**Table 4**).

**Table 4:** Imaging analyses and detection rate of PSMA imaging compared to other imaging techniques and main findings.

Authors	PSMA Analysis	Comparison	Detection Rate (PSMA vs. Comparison)	Main Findings
Rowe et al. (32)	Visual	CE-MRI	100% vs 100%	Intense [18F]DCFPyL uptake (SUVmax 3.9) in a BM previously identified by CE-MRI.
Taywade et al. (17)	Visual	[131I] Scan [18]FDG PET/CT	100% vs 0% 100% vs 20%	Intense [68Ga]PSMA uptake in 5 BMs, With a negative [131I] scan. [18F]FDG PET/CT identified only one.
Medina- Ornelas et al. (30)	Visual	CE-MRI	100% vs 100%	Intense [68Ga]PSMA uptake in a BM progression, found by CE-MRI, confirmed by biopsy. No significant PSMA uptake in other treated BM.
Matsuda et al. (26)	Visual	CE-MRI	100% vs 100%	Heterogeneous, high [89Zr]-Df-IAB2M PET uptake in a BM, confirmed by biopsy and no significant uptake in healthy brain tissue.
Malik et al. (18)	Visual	[18]FDG PET/CT	100% vs 0%	Intense [68Ga]PSMA uptake in a BM that doesn’t have [18]FDG uptake.
Raveenthiran et al. (33)	Visual	CE-CT	100% vs 0%	Intense [68Ga]PSMA uptake in a BM, later confirmed by histopathology.
Filizoglu et al. (34)	Visual	CE-MRI	100% vs 100%	[68Ga]PSMA PET/CT differentiated between RN and progression in BMs, guiding subsequent therapy and confirming disease regression.
Yin et al. (35)	Visual SUVmax	CE-MRI	66% vs 100%	Intense [18F]DCFPyL uptake (SUVmax 3-6) in 2 BMs, equivocal uptake (SUVmax = 0.5) in 1.
Marafi et al. (19)	Visual	CE-MRI [18]FDG PET/CT	100% vs 100% 100% vs 33%	Intense [18F]PSMA in a BM with a mild [18F]FDG uptake and no uptake in normal brain parenchyma or in the post-radiation areas. [18F]PSMA PET/CT detected 2 BMs confirmed by CE-MRI, but [18F]FDG negative.
Hod et al. (37)	Visual	CE-MRI CE-CT	100% vs 100% 100% vs 100%	Intense [68Ga]PSMA uptake in BM, misinterpreted as nonspecific gliosis on CE-MRI. Follow-up CE-CT, CE-MRI and biopsy later confirmed the lesion.
van Boxtel et al. (40)	Visual SUVmax	CE-MRI	100% vs 100%	Homogeneous intense [68Ga]PSMA uptake in BMs (SUVmax of 2.84 ± 3.6).
Pitaula-Cortes et al. (36)	Visual SUVmax TBR	[131I] Scan	100% vs 33%	Higher [68Ga]PSMA uptake in BMs (SUVmax 70.5) than other locations. [131I] scan identified in 1/3 BMs.
Arslan et al. (20)	Visual	CE-MRI [18]FDG PET/CT	100% vs 100% 100% vs 100%	Intense [68Ga]PSMA uptake in a recurrent BM in CE-MRI, confirmed by biopsy, with low [18F]FDG uptake.
Datta Gupta et al. (21)	Visual	CE-MRI [18]FDG PET/CT	100% vs 100% 100% vs 0%	Intense [68Ga]PSMA uptake in a BM, [18F]FDG negative, confirmed by CE-MRI.
Mehdi et al. (38)	Visual SUVmax TBR	CE-MRI	100% vs 100%	Mild [18F]PSMA uptake in BMs, revealed by CE-MRI, but high TBR (pilot study ongoing).
Pei et al. (27)	Visual SUVmax T/L ratio	CE-MRI	100% vs 100%	Intense [68Ga]PSMA uptake in BMs (SUVmax 6-14) compared to primary cancer (SUVmax 2-6) p < 0.01. T/L ratio of 1.76.
Lattuada et al. (28)	Visual SUVmax SUVpeak	CE-MRI	100% vs 100%	Significant difference between BMs and RN uptake on [68Ga]PSMA PET/CT: BMs SUVmax of 7.62 vs 4.05 of RN (p=0.013); BMs SUVpeak 3.71 vs 2.09 of RN (p=0.013).
Dall’Armellina et al. (29)	Visual	CE-MRI	100% vs 100%	Intense [18F]PSMA uptake in BMs; no significant activity in the surrounding CE-MRI-detected edema.
Shamim et al. (22)	Visual SUVmax	[18]FDG PET/CT	100% vs 0%	[68Ga]PSMA uptake in a BM (SUVmax 1.8 ± 0.3), [18]FDG negative.
Andryszak et al. (23)	Visual SUVmax TBR	[18]FDG PET/CT	100% vs 0%	Intense [18F]PSMA uptake (SUVmax of 6 and TBR 59) in a BM, that doesn’t have [18]FDG uptake.
Pruis et al. (31)	Visual SUVmax TBRmax	CE-MRI	100% vs 100%	Intense [68Ga]PSMA uptake in BMs: SUVmax 12-17 (TBR 478-1705) after IV and 128-337 (TBR 10152-30191) after ssIA administration. ssIA led to 15fold higher tumor uptake compared to IV, with no uptake in healthy brain tissue.
Van den Broeck et al. (24)	Visual	CE-MRI [18]FDG PET/CT [131I] Scan	100% vs 100% 100% vs 0% 100% vs 0%	Intense [18F]AlF-PSMA uptake (SUVmax 4-8, MTV 0.44-3.76 ml) in BMs, [18F]FDG and [131I] negative.
Mendanha et al. (39)	Visual	CE-MRI [18]FDG PET/CT	100% vs 100% 100% vs 100%	Higher [18F]PSMA uptake than [18F]FDG PET/CT in BMs, revealed by CE-MRI (SUVmax 8-11).

## 4 Discussion

PSMA-targeted PET/CT is well-established for restaging PCa patients after biochemical recurrence, staging high-risk PCa, and selecting candidates for radioligand therapy (16). The discovery of PSMA glycoprotein overexpression in the endothelial cells of neovascularization in various solid tumors has expanded its potential applications, suggesting that this tracer could also be useful for evaluating a broader range of malignancies (10–12). Its capability to provide high tumor-to-background ratios makes it particularly useful for CNS imaging, where conventional modalities such as MRI and [18F]FDG PET often encounter limitations. This review synthesizes existing evidence to assess PSMA diagnostic utility across various tumor types and its potential integration into neuro-oncological practice.

The quality of the included studies was assessed using the QUADAS-2 tool, revealing variability in risk of bias and applicability concerns. Although case series and case reports might introduce a potential patient selection bias, they were included to broaden the scope of the search, as they provide unique and relevant information not available in systematic studies. The sensitivity analysis highlighted their significant impact on the results, confirming their validity and justifying their inclusion. Nonetheless, the primary aim of this analysis is to evaluate the proof-of-concept studies available in literature and to assess the feasibility of PSMA PET imaging in investigating brain metastases. The index test, PSMA PET, was generally well-performed, although incomplete reporting of imaging protocols in some studies resulted in an unclear risk of bias. While the systematic studies involved two or more independent readers in the analysis of PET images, this was not the case for the case reports and case series. This aspect is acknowledged as a limitation of the review, as it could represent an additional source of bias. No significant differences in the detection rates, diagnostic accuracy or tumor-to-background were observed between different PSMA tracers in brain imaging, indicating that all PSMA tracers could potentially be used in neuro-oncological imaging. The detection rates with PSMA PET were found to be comparable to those with contrast-enhanced MRI, which is currently considered the gold standard for evaluating BMs (7), but the absence of histopathological confirmation in certain cases contributed to a moderate risk of bias. In several cases (17–24), PSMA tracers were able to detect BMs that were not visible with [18F]FDG PET, leading to changes in patient management. Compared to [18F]FDG, PSMA PET exhibits greater diagnostic accuracy, as [18F]FDG shows physiological uptake in the brain that can interfere with detecting small BMs. The low physiological background activity of PSMA leads to a high TBR, making it an effective tool for both diagnostic and potentially therapeutic purposes. Although the results of the review suggest that PSMA PET is more effective than conventional imaging in evaluating BMs, direct comparisons with contrast-enhanced MRI, [18F]FDG PET, or CT are not available for every individual study, thus representing a limitation. Additionally, incomplete follow-up or lack of clarity in the timing between the index test and reference standard led to an unclear risk of bias in several studies. These findings emphasize the need for standardized methodologies and prospective studies to validate the diagnostic accuracy of PSMA PET in detecting brain metastases. Ongoing clinical trials are exploring the use of PSMA tracers for both diagnostic and theranostic applications in malignant brain tumors (ClinicalTrials.gov ID: NCT05798273, NCT06241391, NCT06209567). PSMA-targeted radioligands, when paired with alpha- or beta-emitting radionuclides, have shown promise in the treatment of advanced metastatic prostate cancer (25), suggesting that similar therapies could be beneficial for brain metastases, which are often associated with neovascularization and PSMA overexpression.

### 4.1 Tumor-specific insights

#### 4.1.1 Lung cancer

PSMA PET has demonstrated a high degree of tracer uptake in brain metastatic lesions compared to primary tumors, suggesting that lung cancer metastases may exhibit unique PSMA expression profiles. Matsuda et al. (26) described a case of lung cancer where [89Zr]DfIAB2M (anti-PSMA minibody that binds the extracellular domain of PSMA) PET/CT revealed high heterogeneous PSMA uptake in a brain lesion detected by CE-MRI, with no significant uptake in healthy brain tissue. Immunohistochemistry showed moderate PSMA expression in the regions corresponding to areas with high or moderate uptake on the PSMA PET scan. Similarly, Pei et al. (27) documented a study involving seven lung cancer patients (six Non-Small Cell Lung Cancer and one Small Cell Lung Cancer) with BMs. They found significantly higher [68Ga]PSMA-11 uptake in the metastases than in the primary lung cancers: SUVmax of primary lung cancer ranged from 1.8 to 5.6, while in the BMs they ranged from 5.6 to 13.8 (P < 0.01). On average, the SUVmax in metastases was 1.76 times higher than in the liver (T/L ratio), suggesting the potential for PSMA RLT to detect metastatic sites. Lattuada et al. (28) presented findings on the use of [68Ga]PSMA PET/CT to differentiate BMs from lung from RN. The study analyzed 37 lesions, of which 15 were BMs (mean SUVmax of 7.62; 95% CI 5.10 - 10.15) and 22 were RN (mean SUVmax 4.05; 95% CI 2.98 - 5.12), with a statistically significant difference. The mean SUVpeak for the BMs group was 3.71 (95% CI 2.40 - 5.01), while for the RN group it was 2.09 (95% CI 1.57 - 2.61), with a statistically significant difference. The conclusion highlighted a significant difference in PSMA radioligand between BMs and RN, supporting the potential of [68Ga]PSMA PET/CT for distinguishing these two conditions.

Dall’Armellina et al. (29) reported a case of a patient with a synchronous diagnosis of high-risk PCa and Non-Small Cell Lung Cancer. A [18F]PSMA-1007 PET/CT scan revealed two areas of abnormal uptake in the brain, located in the left frontal and temporal lobes. Intense, focal tracer uptake was observed exclusively in the brain lesions, while the surrounding edema, visible on MRI, showed no significant activity. However, there is a significant difference in the biological characteristics between small cell lung cancer and non-small cell lung cancer, particularly regarding aggressiveness and the tendency to metastasize. Currently, the available data on the use of PSMA PET in these two subtypes is limited, making it necessary to develop specific trials to better understand the differences in PSMA expressions both in the primary tumor and in metastases, as well as its potential clinical impact in each of these neoplasms.

#### 4.1.2 Breast cancer

Medina-Ornelas et al. (30) presented a case report of a woman with HER-2neu positive breast carcinoma, initially treated with neoadjuvant chemotherapy and radical mastectomy for infiltrating ductal carcinoma (T2N1M0), who developed neurological symptoms four months later. MRI revealed two brain lesions, and further radiotherapy and chemotherapy were administered. Despite treatment, a [68Ga]PSMA PET/CT revealed intense uptake in one BM, confirmed to have progressed via CE-MRI and biopsy. No significant PSMA uptake was found at the site of the additional previously treated and healed BM. Malik et al. (18) and Marafi et al. (19) demonstrated the effectiveness of PSMA-targeted PET/CT in detecting breast cancer BMs during restaging, particularly in cases where [18F]FDG PET/CT showed minimal or no uptake. In contrast, PSMA PET/CT successfully highlighted active BMs, emphasizing its utility in identifying lesions traditional FDG-based scans might miss. Furthermore, in Marafi et al. case (19), a woman with triple-negative breast cancer who had undergone gamma knife radiotherapy for a BM was later evaluated with both [18F]FDG and [18F]PSMA-1007 PET/CT. While the [18F]FDG PET/CT scan showed uptake in both the normal brain parenchyma and the post-radiation areas, [18F]PSMA-1007 PET/CT did not exhibit uptake in these regions. This finding highlights its usefulness in distinguishing recurrence from RN in post-treatment evaluations. Arslan et al. (20) presented another case of a woman with triple-negative breast cancer. Thirteen months after initial treatment, a recurrence of BMs showed high PSMA uptake but only mild FDG uptake. In a prospective study by Andryszak et al. (23), [18F]PSMA-1007 PET/CT identified ten small BMs in a patient with triple-negative breast cancer undergoing palliative chemotherapy, which were [18F]FDG negative but later confirmed by MRI. The lesions were 4-7 mm in diameter, with a PSMA SUVmax of 5.9 and a TBR of 59. Pruis et al.’s prospective study (31) involved five patients with BMs, one from breast cancer and four from lung cancer, using PET/CT following both super-selective intra-arterial (ssIA) and intravenous (IV) administration. In all patients, [68Ga]PSMA-11 uptake in the brain corresponded to areas of contrast enhancement seen on MRI. In the breast cancer patient, the BM identified during staging showed an SUVmax of 13 after IV administration compared to 215 following ssIA, with a tumor-to-background ratio (TBR) of 1311 versus 10152. For the three lung cancer patients in restaging, BMs detected showed SUVmax values ranging from 12 to 17 with IV administration and from 128 to 288 with ssIA, along with TBR values of 1196-1705 (IV) versus 2096-12880 (ssIA). In conclusion, ssIA administration led to a 15-fold higher tumor uptake than IV administration, with negligible uptake in healthy brain tissue, potentially expanding the number of patients eligible for radioligand therapy (RLT).

#### 4.1.3 Renal cancer

Numerous cases of significant PSMA tracer uptake from renal tumors have also been observed in BMs. Rowe et al. (32) studied five patients with renal cell carcinoma using [18F]DCFPyL PET/CT and CE-MRI, identifying one patient with BMs. This patient had previously undergone a nephrectomy and was diagnosed with clear cell renal cell carcinoma, without any prior systemic therapy. A CE-MRI revealed a brain lesion in the left frontal lobe, which showed intense [18F]DCFPyL uptake, with an SUVmax of 3.9. Similarly, Raveenthiran et al. (33) identified unknown BMs in a patient with clear cell renal cell carcinoma through [68Ga]PSMA-11 PET/CT. The presence of the BM was later confirmed via histopathology, leading to a modification in the patient’s treatment plan. Filizoglu et al. (34) reported the case of a patient with a history of clear cell renal cell carcinoma who developed two BMs and was treated with stereotactic surgery and whole-brain irradiation alongside concurrent nivolumab. During follow-up, CE-MRI suggested possible RN after the stereotactic radiosurgery, but [68Ga]PSMA PET/CT revealed disease progression instead. After further brain therapy, [68Ga]PSMA PET/CT indicated disease regression, consistent with the CE-MRI findings. Yin et al. (35) explored the application of [18F]DCFPyL PET/CT in patients with metastatic non-clear cell renal cell carcinoma, identifying one patient with three BMs. In this case, the median SUVmax ranged from 0.5 to 6.2, with two lesions showing significant uptake (SUVmax 3.4 and 6.2) and one displaying equivocal uptake (SUVmax 0.5). The authors noted that it remains unclear whether some lesions with equivocal uptake may reflect the effects of previous treatments.

#### 4.1.4 Thyroid cancer

Radioiodine-refractory thyroid cancer remains a diagnostic challenge, with conventional [131I] scintigraphy often failing to detect BMs. In this context, PSMA PET has demonstrated superior sensitivity in this context, particularly in identifying metastases with low iodine uptake. Taywade et al. (17) presented a patient who underwent total thyroidectomy, left neck dissection, and subsequent [131I] therapy for cervical lymph node recurrence. Following an increase in thyroglobulin levels and a negative [131I] scan, the patient was evaluated using both [68Ga]PSMA-11 and [18F]FDG PET/CT. The PSMA scan detected five BMs, while the FDG scan identified only one. Pitaula-Cortes et al. (36) conducted a retrospective study comparing [68Ga]PSMA-11 PET/CT and post-therapeutic [131I] whole-body scans with complementary SPECT/CT in 10 patients with well-differentiated metastatic thyroid cancer. Among these patients, three BMs from papillary thyroid carcinoma were identified in two patients. All BMs showed PSMA avidity, while only one had significant [131I] uptake. Notably, the highest PSMA uptake among all disease sites was in the BMs (SUVmax 70.5, with a TBR of 74). In a prospective study, Van den Broeck et al. (24) evaluated the potential application of [18F]AlF-PSMA-11 in patients with radioiodine-refractory thyroid carcinoma. Out of 8 patients, 2 had single BMs, each showing [18F]AlF-PSMA-11 uptake. Neither metastasis showed uptake with [18F]FDG.

#### 4.1.5 Melanoma

A few reports have highlighted cases of melanoma BMs exhibiting PSMA uptake. One case report by Hod et al. (37) describes a patient who underwent [68Ga]PSMA PET/CT for PCa, which unexpectedly revealed brain uptake at the site of a previously treated melanoma metastasis. Initially, the finding was deemed nonspecific and misinterpreted as gliosis resulting from surgery and radiation on CE-MRI. However, six months later, follow-up CE-CT and MRI scans confirmed a recurrence at the same location, which was subsequently histopathologically validated. A pilot study by Mehdi et al. (38) explored the feasibility and utility of [18F]PSMA-1007 PET/CT in managing brain tumors, including a case of multiple melanoma BMs that exhibited mild [18F]PSMA-1007 uptake while still showing a high TBR. Additionally, a case report by Mendanha et al. (39) detailed a patient with acral melanoma who had undergone amputation and later developed multiple BMs. A subsequent [18F]PSMA-1007 PET/CT scan demonstrated high uptake in all BMs (SUVmax ranging from 8 to 11), which had only mild uptake on [18F]FDG PET/CT.

#### 4.1.6 Salivary gland cancer

BMs originating from salivary gland tumors have also shown significant uptake of PSMA-targeting tracers. In a prospective study by Van Boxtel et al. (40), a [68Ga]PSMA-11 PET/CT scan was performed on 25 patients with salivary gland cancer. One patient with salivary duct carcinoma, who had not received androgen deprivation therapy, was found to have 13 BMs on CE-MRI, all demonstrating homogeneous PSMA uptake (SUVmax 2.84 ± 3.6). Datta Gupta et al. (21) reported a case involving a BM from adenoid cystic carcinoma of the right parotid gland. The patient had previously undergone surgery, local radiotherapy, and chemotherapy. While an [18F]FDG PET/CT scan did not reveal any abnormal tracer accumulation in the brain, a [68Ga]PSMA PET/CT scan indicated focal uptake in the right cerebellum, later confirmed by CE-MRI as BM. Lastly, Shamim et al. (22) documented two additional cases of BM from adenoid cystic carcinoma exhibiting [68Ga]PSMA-11 uptake (SUVmax 1.8 ± 0.3), which was not visible on [18F]FDG PET/CT in a prospective study.

### 4.2 Potential for theranostics

While existing studies’ primary focus has been diagnostic applications, PSMA stands out as an excellent target for radioligand therapy, demonstrating significant potential for theranostic use. Radiopharmaceuticals paired with therapeutic radionuclides, such as lutetium-177 or actinium-225, offer a dual diagnostic and therapeutic approach. Preliminary studies and case reports have demonstrated mixed results regarding the use of PSMA-based radiopharmaceuticals in therapy for non-prostatic cancers, including brain tumors (13–15, 41–43). One clinical trial is currently investigating the dosimetry and immunohistochemistry of PSMA radiolabeled agents as potential therapeutic targets in glioma treatment (NCT05263466). Additionally, a Phase I/II clinical trial (ClinicalTrials.gov ID: NCT05278208) is assessing the effectiveness of [177Lu]Lu-DOTATATE for treating recurrent or progressive high-grade CNS tumors.

The use of PSMA as a treatment for non-prostatic cancers is still in the exploratory phase, and its potential benefits have yet to be clearly defined for each type of neoplasm. Current studies employing these tracers in the neuro-oncological field primarily focus on primary tumors, particularly in gliomas, overlooking brain metastases originating from non-prostatic neoplasms. These investigations must first face and overcome the challenge of demonstrating a positive response in primary brain tumors, thereby paving the way for a broader indication of these therapies in the treatment of non-prostatic brain metastases. Expanding theranostic applications to non-prostatic tumors has the potential to revolutionize the treatment landscape, particularly in neuro-oncology. However further clinical research is needed to explore the therapeutic potential and dosimetric considerations in greater detail to optimize the management of brain metastases. It is also critical to better understand radiotoxicity and the effects of irradiation on surrounding brain tissue to ensure the safety and efficacy of these treatments.

### 4.3 Challenges

Despite its promise, PSMA PET imaging faces several limitations. Tumor-specific variability in PSMA expression may result in inconsistent uptake patterns, necessitating validation through larger, multicenter studies. We believe that PSMA PET could serve as a complementary tool in neuro-oncological workflows, particularly in cases where contrast-enhanced MRI as the current standard for evaluating brain metastases, yields inconclusive results. This approach would enable the assessment of intertumoral heterogeneity in PSMA expression across different tumor histotypes, helping to determine its clinical applicability on a case-by-case basis. False positives, such as uptake in inflammatory conditions, and false negatives in small lesions below spatial resolution, highlight the need for complementary imaging modalities (44). Additionally, logistical barriers, such as limited availability of PSMA-targeted tracers and their production requirements, may restrict widespread clinical adoption (45, 46). To expand the clinical indications of PSMA PET beyond prostate cancer, it would be essential to establish a network of PET centers dedicated to its use. Such an infrastructure would not only enable the collection of larger, multicenter patient cohorts for research but also facilitate the integration of PSMA PET into other fields, including neuro-oncology.

### 4.4 Future directions

Early studies have shown promising results for using PSMA-based radioligand therapy (RLT) in treating non-prostatic solid tumors. However, prospective clinical trials with larger patient cohorts are essential to fully explore its applicability and efficacy in neuro-oncology. Further studies are also needed to confirm the role of PSMA-targeted imaging for other solid tumors and assess its impact on treatment outcomes, particularly in brain metastases. PSMA-guided imaging could also play a crucial role in assessing responses to treatments, especially radiotherapy. In several cases, PSMA imaging has demonstrated the ability to differentiate between radiation necrosis and the persistence or recurrence of disease, suggesting its potential as a valuable tool in monitoring therapeutic outcomes (19, 28, 34). Additionally, PET with PSMA tracers could be used in neuro-oncology to guide and optimize radiotherapy administration, improving the accuracy of treatment and the precision of tumor localization.

## 5 Conclusions

PSMA-targeted tracers have shown great potential in imaging brain metastases from non-prostatic solid tumors. However, further clinical studies are essential to confirm their efficacy and optimize their use in neuro-oncology. Comparative evaluations with current reference imaging methods and the establishment of clinical recommendations will be crucial for integrating PSMA PET into routine neuro-oncological care.

## Data Availability

The original contributions presented in the study are included in the article/supplementary material. Further inquiries can be directed to the corresponding author.
